# Supplementary material for: Molecular Glues Stabilize Water‐Mediated Hydrogen Bonds in Ternary Complexes
Source: Chemphyschem. 2026 Jan 25;27(2):e202500765. doi: 10.1002/cphc.202500765 (PMC12833552; doi:10.1002/cphc.202500765)
Supplement: Supplementary file 1 — Supplementary Material [file CPHC-27-e202500765-s001.pdf]

Molecular glues stabilize water-mediated hydrogen bonds in ternary complexes

Apoorva Mathur[a], Mariona Alegre Canela[a,b], Max von Graevenitz[a], Chiara Gerstner[a], Ariane Nunes-Alves[a,*]

a A. Mathur, M.A. Canela, M. v. Graevenitz, C. Gerstner, A. Nunes-Alves
Institute of Chemistry, Technische Universität Berlin, Straße des 17. Juni 135, 10623 Berlin, Germany
E-mail: ferreira.nunes.alves@tu-berlin.de

b M.A. Canela
Barcelona Tech, Universitat Politècnica de Catalunya, Carrer de Jordi Girona, 31, Les Corts, 08034 Barcelona, Spain

1 Methods

1.1 System setup

We chose two ternary complexes for the study, FKBP12-FRAP-rapamycin (PDB ID 1FAP[1]) and FKBP12-CEP250-WDB002 (PDB ID 6OQA[2]). While the FKBP12-FRAP-rapamycin complex structure has one unit of each molecule (FKBP12, FRAP and rapamycin), the FKBP12-CEP250-WDB002 complex structure contains two units of each molecule (FKBP12, CEP250 and WDB002), with two long alpha helical strands of CEP250 coiled around each other and two FKBP12-WDB002 complexes, bound on either side. The crystallographic waters were preserved for the MD simulations, since they may be involved in important water-mediated hydrogen bonds. Protonation states of the protein residues were assigned using PDB2PQR[3?] with the PROPKA method according to the pH value of the respective crystallization experiment, i.e., pH 8 for FKBP12-FRAP-rapamycin and pH 7.4 for FKBP12-CEP250-WDB002. To study the effects of molecular glues on the ternary complexes, simulations of the complexes with the molecular glue (holo complexes) and without the molecular glue (apo complexes) were performed. In addition, simulations of the binary protein-molecular glue complexes were performed for molecular mechanics / generalized Born surface area (MM/GBSA) binding free energy calculations.

1.2 Molecular dynamics simulations

In order to parameterize the molecular glues, rapamycin and WDB002, the partial charges of the atoms were determined using quantum mechanical (QM) calculations and the restrained electrostatic potential (RESP) method [4]. The input molecular glue conformations were obtained from the crystal structures retrieved from RCSB PDB. The molecular glues were first protonated using OpenBabel [5], followed by a QM calculation performed using Gaussian09 [6] at the HF/6-31G* level to obtain their electrostatic potentials. These potentials were then used to calculate the RESP partial charges. Finally, the general AMBER force field (GAFF) [7] was used to determine the bond, angle, torsion, and van der Waals (vdW) parameters for the molecular glues. The protein was described using the AMBER-ILDN force field [8]. The TIP3P [9] water model was used to solvate the systems, with a padding of 10 Å. The systems were neutralized using Na+ and Cl- ions at the physiological concentration of 150 mM.

Three replica simulations were performed for each system using GROMACS 2020.1 for FKBP12-FRAP-rapamycin and GROMACS 2024.2 for FKBP12-CEP250-WDB002 systems. The steepest descent method was used to perform energy minimization for 5000 steps for each system using positional restraints of 1000 $kJ/mol/nm^2$ on the heavy atoms of the protein and molecular glues. Then, the systems were heated to 300 K in the NVT ensemble using the Berendsen thermostat. Next, the pressure was equilibrated to 1 bar using the Berendsen barostat for pressure coupling with positional restraints applied. The positional restraints were then gradually reduced (500, 100, 10 $kJ/mol/nm^2$) in 500 ps simulations. Production runs of 500 ns were performed with a timestep of 2 fs. In the production runs, the temperature coupling was realized with the Nose-Hoover thermostat and a time constant of 0.1 ps, maintaining a reference temperature of 300 K, and pressure coupling was achieved with the Parrinello-Rahman barostat with a compressibility of $4.5*10^{-5}$ bar^{-1} and a time constant of 5 ps, maintaining a reference pressure of 1 bar. LINCS [10] and SETTLE [11] algorithms were used to constrain covalent bonds with hydrogens and solvent bond lengths, respectively. Particle mesh Ewald (PME) [12] was used to calculate the electrostatic interactions with a real-space cutoff of 1.2 nm, PME order of four, and a Fourier grid spacing of 1.2 Å. A cut-off of 1.2 nm was used for the calculation of the van der Waals interactions.

1.3 Computation of binding free energies

Binding free energies were calculated using the MM/GBSA method as implemented in AMBER [13], based on the following equation:

$$\Delta G_{\text{bind}} = G_{\text{complex}} - (G_{\text{receptor}} + G_{\text{ligand}}) \tag{1}$$

where ΔG_{bind} represents the binding free energy, and G represents the free energy of the respective state (complex, receptor, or ligand). For ternary complexes, the receptor could be a protein or a protein-molecular glue complex, and

the ligand could be the molecular glue or a protein. The free energy decomposition is given by:

$$\Delta G_{\text{bind}} = \Delta E_{\text{bonded}} + \Delta E_{\text{non-bonded}} + \Delta G_{\text{solv}} - T\Delta S \qquad (2)$$

In practice, the binding free energy was calculated as:

$$\Delta G_{\text{bind}} = \Delta E_{\text{vdw}} + \Delta E_{\text{elec}} + \Delta G_{\text{GB}} + \Delta G_{\text{SA}} \qquad (3)$$

where ΔE_{bonded} and $\Delta E_{\text{non-bonded}}$ are the change in bonded and non-bonded interaction energies for solutes upon complex formation, respectively, T is the temperature, S is the entropy, and ΔG_{solv} is the change in solvation energy upon complex formation. ΔE_{vdw} represents the change in van der Waals interaction energies, ΔE_{elec} represents the change in electrostatic interaction energies, and ΔG_{GB} and ΔG_{SA} represent the generalized Born (GB) term and surface area (SA) term, respectively. GB and SA represent the changes in polar and nonpolar solvation energies. ΔE_{bonded}, the bond, angle, and dihedral energies, are zero in MM/GBSA when the same trajectory is used to calculate $G_{complex}$, $G_{receptor}$ and G_{ligand}, as done here. The entropic contribution (-TΔS) can be approximated using normal mode analysis or quasi-harmonic approximation. However, it is usually omitted due to high computational cost, and it was not used in our calculations. Energy terms to compute MM/GBSA binding free energies were calculated using every 10th frame from MD simulations.

To assess the favourability of the interaction between the FKBP12-molecular glue complex with the target protein (FRAP or CEP250), the binding free energy of the ternary complex (FKBP12-molecular glue-target protein) was compared with that of the binary complexes (FKBP12-molecular glue and target protein-molecular glue) [14,15].

1.4 Data analysis

Root mean square deviation (RMSD), root mean square fluctuation (RMSF), pairwise distances and the number of hydrogen bonds (H-bonds) between FKBP12 and the target protein were calculated using GROMACS modules [16]. Water-mediated H-bonds were identified using MD Analysis with 3 Å as distance cutoff between donor and acceptor atoms of residues or water and 120º as angle cutoff [17]. Only interactions which lasted for at least 20% of the simulation length are shown in the paper.

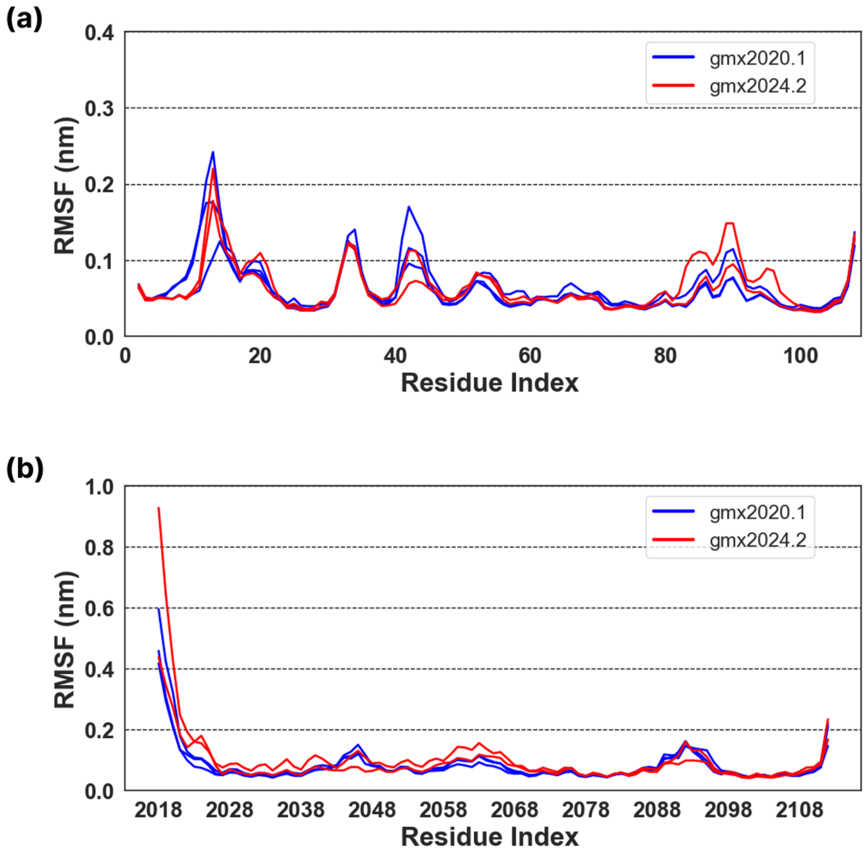

Figure S1: Root mean square fluctuations of (a) FKBP12 and (b) FRAP in the holo FKBP12-FRAP-rapamycin complex, simulated with GROMACS 2020.1 (3 replicas of 500 ns) or GROMACS 2024.2 (2 replicas of 500 ns) , show that the fluctuations of the proteins are similar in both versions of GROMACS.

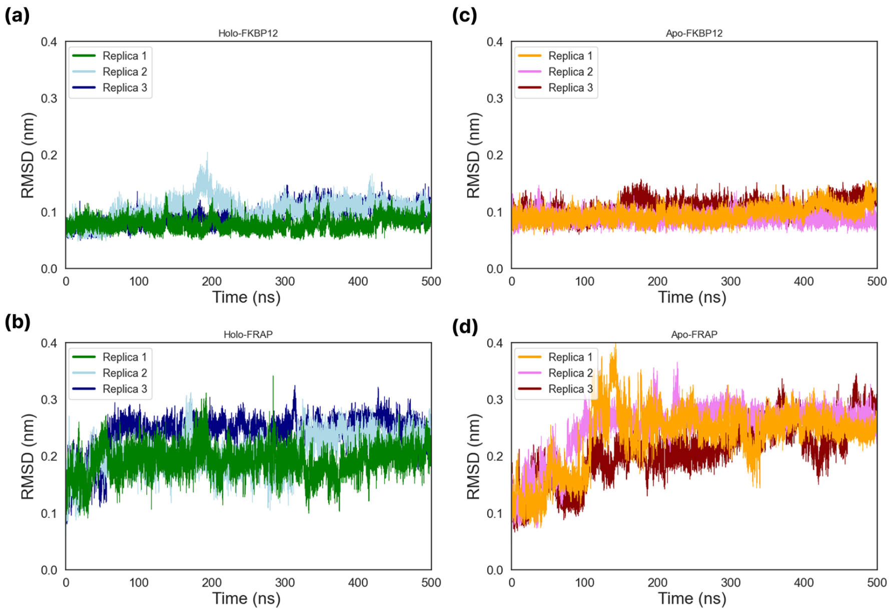

Figure S2: Root mean square deviation (RMSD) of backbone atoms after alignment with the backbone of FKBP12 (top row) and FRAP (bottom row) in holo (a, b) and apo (c, d) conditions from three replica MD simulations of 500 ns.

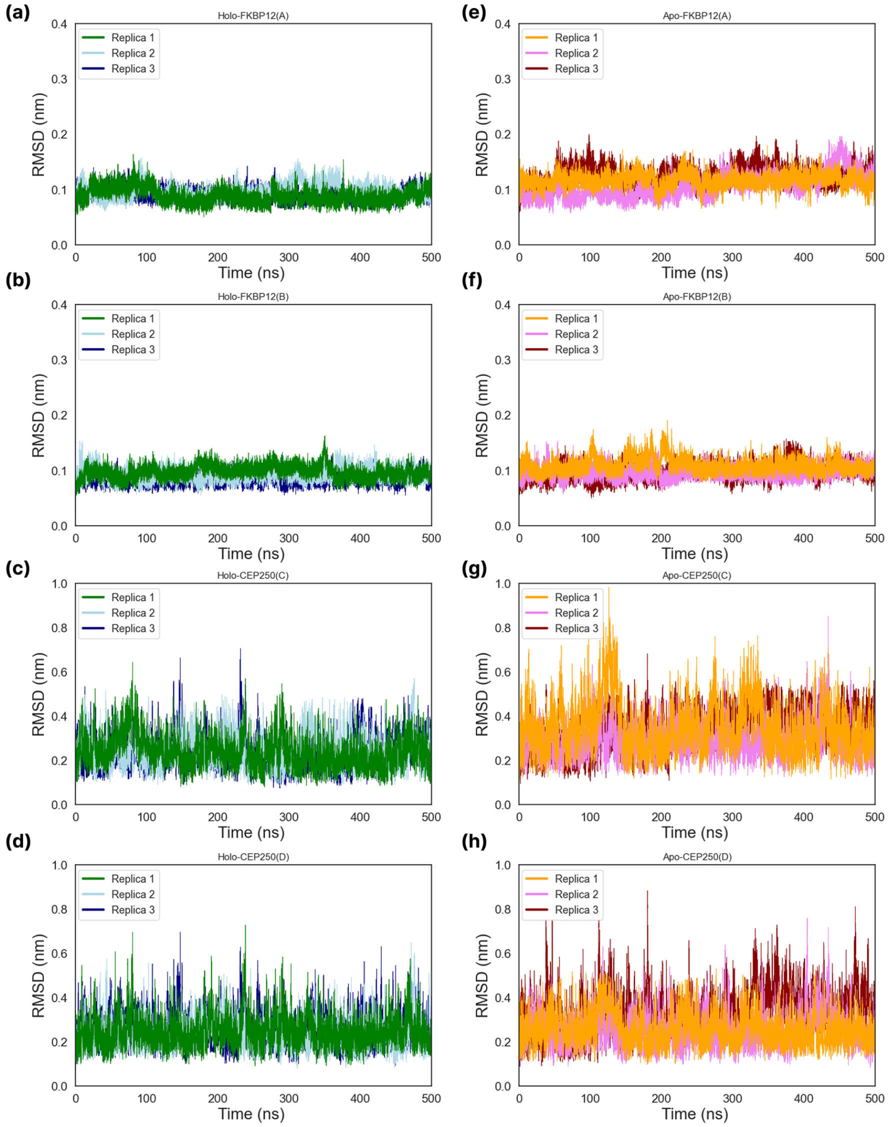

Figure S3: Root mean square deviation (RMSD) of backbone atoms after alignment with the backbone of FKBP12 (chain A, 1st row), FKBP12 (chain B, 2nd row), CEP250 (chain C, 3rd row) and CEP250 (chain D, 4th row) in holo (a-d) and apo (e-h) conditions from three replica MD simulations of 500 ns.

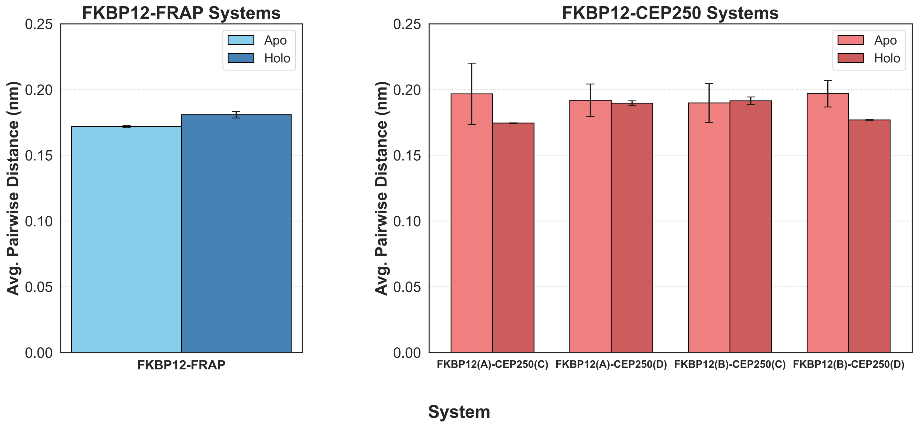

Figure S4: Pairwise minimum distances between FKBP12 and FRAP, and between FKBP12 (chain A or B) and CEP250 (chain B or C), averaged over three replica simulations of 500 ns in apo and holo conditions. The proteins are closer by a few Ångströms in the apo condition for the FKBP12-FRAP-rapamycin complex, in comparison to the holo condition.

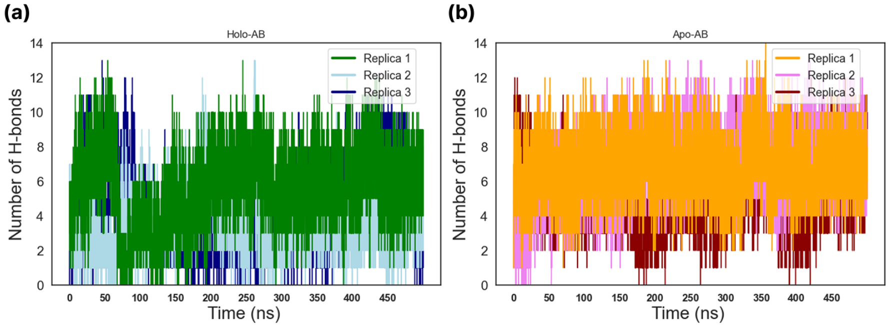

Figure S5: Total number of hydrogen bonds between FKBP12 and FRAP in holo (a) and apo (b) conditions from three replica MD simulations of 500 ns.

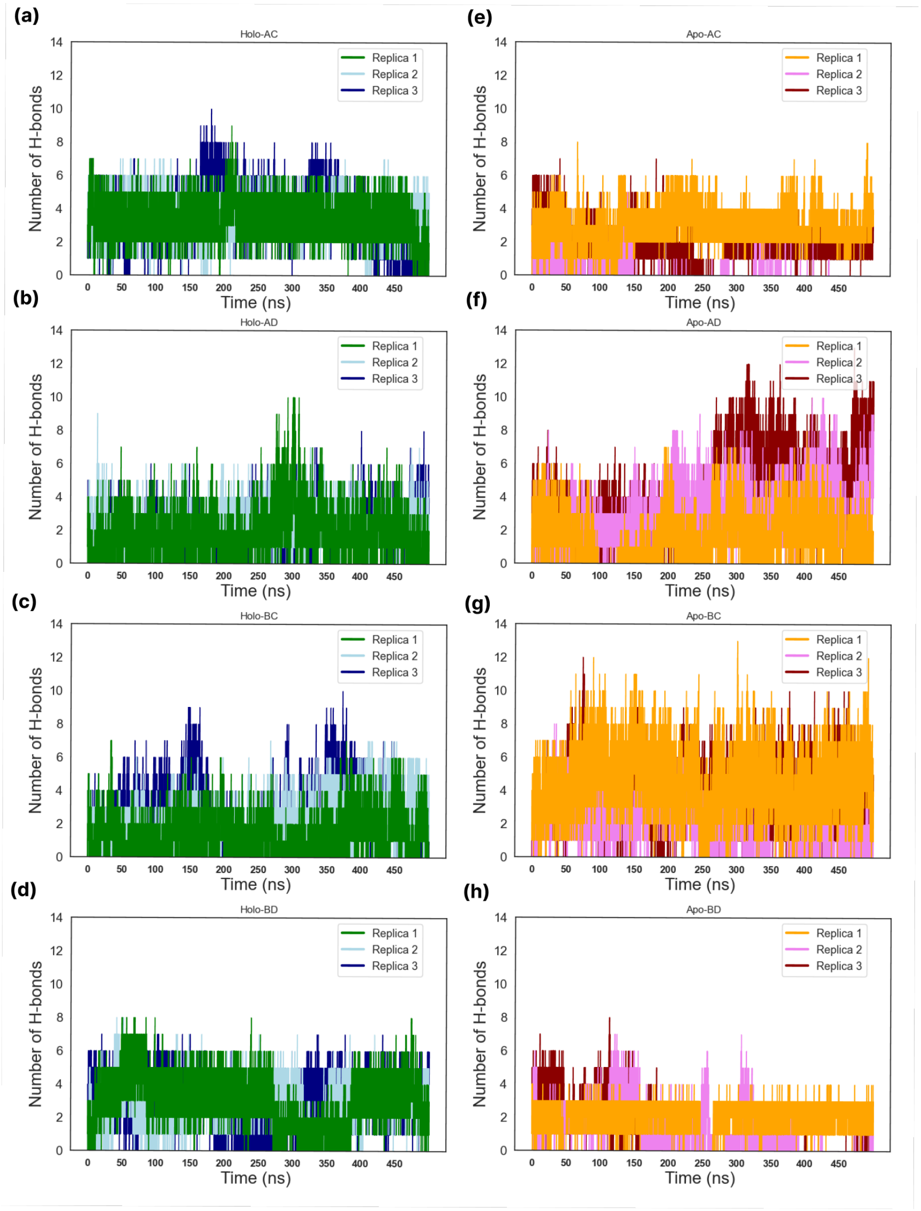

Figure S6: Total number of hydrogen bonds between FKBP12 and CEP250 in holo (a-d) and apo (e-h) conditions from three replica MD simulations of 500 ns.

Table S1: Summary of binding free energies computed using molecular mechanics / generalized Born surface area (MM/GBSA), or computed from experimentally determined dissociation constants (K_d) for FKBP12-FRAP-rapamycin[18] and FKBP12-CEP25-WDB002[2].

**Receptor**	**Ligand**	**Experimental K_d (nM)**	**Experimental binding free energy (kcal/mol)**	**MM/GBSA binding free energy (kcal/mol)**
FKBP12	Rapamycin	0.2	-13.21	-49.48 ± 3.95
FRAP	Rapamycin	26,000	-6.25	-34.82 ± 0.70
FKBP12-Rapamycin	FRAP	12	-10.79	-61.15 ± 5.30
FRAP-Rapamycin	FKBP12	1×10^{-4}	-17.7	-93.26 ± 5.55
FKBP12	WDB002	5.2 ± 0.4	-11.29 ± 0.05	-50.26 ± 2.72
CEP250	WDB002	NA [a]	NA [a]	-20.82 ± 0.70
FKBP12-WDB002	CEP250	41.2 ± 5.7	-10.07 ± 0.08	-63.34 ± 1.66

[a] NA: Information not available.

Table S2: Summary of residue pairs participating in water-mediated hydrogen bonds in FKBP12-FRAP-rapamycin and FKBP12-CEP250-WDB002 (the same residue pairs are shown in figures 4 and 5).

**FKBP12-FRAP-rapamycin**		**FKBP12-CEP250-WDB002**	
FKBP12	FRAP	FKBP12	CEP250
		F37	Q2191
D38	T2098	D38	Q2191
		S39	Q2191
R43	Q2099, D2102,	R43	S2188, Q2191
K45	D2102		
K48	Y2105, R2109	K48	Q2198
		K53	D2205
E55	Y2105, R2109	E55	R2204
Y83	R2042	Y83	Q2198
G87	R2042		
H88	Y2038	H88	S2189, Q2198
		P89	S2189
		G90	Q2191

References

[1] J. Choi, J. Chen, S. L. Schreiber, J. Clardy, *Science* **1996**, *273*, 239.

[2] U. K. Shigdel, S.-J. Lee, M. E. Sowa, B. R. Bowman, K. Robison, M. Zhou, K. H. Pua, D. T. Stiles, J. A. Blodgett, D. W. Udwary, et al., *Proceedings of the National Academy of Sciences* **2020**, *117*, 17195.

[3] T. J. Dolinsky, J. E. Nielsen, J. A. McCammon, N. A. Baker, *Nucleic Acids Research* **2004**, *32*, W665.

[4] C. I. Bayly, P. Cieplak, W. Cornell, P. A. Kollman, *The Journal of Physical Chemistry* **1993**, *97*, 10269.

[5] N. M. O'Boyle, M. Banck, C. A. James, C. Morley, T. Vandermeersch, G. R. Hutchison, *Journal of Cheminformatics* **2011**, *3*, 33.

[6] M. J. Frisch, G. W. Trucks, H. B. Schlegel, G. E. Scuseria, M. A. Robb, J. R. Cheeseman, G. Scalmani, V. Barone, G. A. Petersson, H. Nakatsuji, X. Li, M. Caricato, A. Marenich, J. Bloino, B. G. Janesko, R. Gomperts, B. Mennucci, H. P. Hratchian, J. V. Ortiz, A. F. Izmaylov, J. L. Sonnenberg, D. Williams-Young, F. Ding, F. Lipparini, F. Egidi, J. Goings, B. Peng, A. Petrone, T. Henderson, D. Ranasinghe, V. G. Zakrzewski, J. Gao, N. Rega, G. Zheng, W. Liang, M. Hada, M. Ehara, K. Toyota, R. Fukuda, J. Hasegawa, M. Ishida, T. Nakajima, Y. Honda, O. Kitao, H. Nakai, T. Vreven, K. Throssell, J. A. Montgomery Jr., J. E. Peralta, F. Ogliaro, M. Bearpark, J. J. Heyd, E. Brothers, K. N. Kudin, V. N. Staroverov, T. Keith, R. Kobayashi, J. Normand, K. Raghavachari, A. Rendell, J. C. Burant, S. S. Iyengar, J. Tomasi, M. Cossi, J. M. Millam, M. Klene, C. Adamo, R. Cammi, J. W. Ochterski, R. L. Martin, K. Morokuma, O. Farkas, J. B. Foresman, D. J. Fox, *Gaussian 09, Revision A.02*, Gaussian, Inc., Wallingford, CT **2016**, http://gaussian.com.

[7] J. Wang, R. M. Wolf, J. W. Caldwell, P. A. Kollman, D. A. Case, *Journal of Computational Chemistry* **2004**, *25*, 1157.

[8] K. Lindorff-Larsen, S. Piana, K. Palmo, P. Maragakis, J. L. Klepeis, R. O. Dror, D. E. Shaw, *Proteins: Structure, Function, and Bioinformatics* **2010**, *78*, 1950.

[9] W. L. Jorgensen, J. Chandrasekhar, J. D. Madura, R. W. Impey, M. L. Klein, *The Journal of Chemical Physics* **1983**, *79*, 926.

[10] B. Hess, H. Bekker, H. J. Berendsen, J. G. Fraaije, *Journal of Computational Chemistry* **1997**, *18*, 1463.

[11] S. Miyamoto, P. A. Kollman, *Journal of Computational Chemistry* **1992**, *13*, 952.

[12] T. Darden, D. York, L. Pedersen, et al., *Journal of Chemical Physics* **1993**, *98*, 10089.

[13] M. S. Valdés-Tresanco, M. E. Valdés-Tresanco, P. A. Valiente, E. Moreno, *Journal of Chemical Theory and Computation* **2021**, *17*, 6281.

[14] B. G. AS, D. Agrawal, N. M. Kulkarni, R. Vetrivel, K. Gurram, *ACS omega* **2024**, *9*, 12611.

[15] W. Li, J. Zhang, L. Guo, Q. Wang, *Journal of Chemical Information and Modeling* **2022**, *62*, 523.

[16] M. Abraham, A. Alekseenko, C. Bergh, C. Blau, E. Briand, M. Doijade, S. Fleischmann, V. Gapsys, G. Garg, S. Gorelov, et al., *GROMACS: Groningen, The Netherlands* **2023**.

[17] R. J. Gowers, M. Linke, J. Barnoud, T. J. E. Reddy, M. N. Melo, S. L. Seyler, J. Domanski, D. L. Dotson, S. Buchoux, I. M. Kenney, et al., MDAnalysis: a Python package for the rapid analysis of molecular dynamics simulations, Technical report, Los Alamos National Laboratory (LANL), Los Alamos, NM (United States) **2019**.

[18] L. A. Banaszynski, C. W. Liu, T. J. Wandless, *Journal of the American Chemical Society* **2005**, *127*, 4715.
